# Association of Autonomic Balance With Phone Call Duration in Healthy Individuals

**DOI:** 10.7759/cureus.33566

**Published:** 2023-01-09

**Authors:** Vibha Gangwar, Shweta Gupta, Manish Verma, Arvind Kumar Singh, Nitin John, Rajani Bala Jasrotia, Amita Singh

**Affiliations:** 1 Department of Physiology, Dr. Ram Manohar Lohia Institute of Medical Sciences, Lucknow, IND; 2 Department of Physiology, Prasad Institute of Medical Sciences, Lucknow, IND; 3 Department of Community Medicine, Dr. Ram Manohar Lohia Institute of Medical Sciences, Lucknow, IND; 4 Department of Physiology, All India Institute of Medical Sciences, Bibinagar, Hyderabad, IND; 5 Department of Physiology, All India Institute of Medical Sciences, Deoghar, Deoghar, IND

**Keywords:** blood pressure, mobile phone, heart rate variability, electromagnetic field, covid-19

## Abstract

Background

This study aimed to estimate the association of autonomic balance with the duration of phone calls in healthy individuals.

Methodology

A total of 30 subjects aged between 18 and 30 years without any established systemic disease and using mobile phones for more than five years with minimum daily usage of 30 minutes were included in this analytical study. Heart rate variability (HRV) was recorded using a three-channel physiograph (AD Instruments South Asia (India) Pvt. Ltd., New Delhi, India) with the software LabChart PROV8.1.8 with HRV Module version 2.0.3 for 10 minutes. Time domain parameters were recorded in terms of the standard deviation of normal to normal interval (SDNN), root mean square of successive differences between normal heartbeats (RMSSD), R-R intervals greater than 50 ms (pRR50), and mean heart rate (MHR), and frequency domain parameters were total power, low-frequency power (LF), high-frequency power (HF), and the ratio of low-frequency to high-frequency power (LF/HF). HRV was recorded three times in each subject that included baseline HRV, HRV during the use of a mobile phone, and HRV after the use of a mobile phone.

Results

A total of 30 subjects (14 males and 16 females) participated in this study. The mean age of participants was 31.93 ± 8.59 years (32.07 ± 9.87 years for males, and 31.81 ± 7.64 years for females). There were no findings of significant arrhythmia in any of the participants. There was a significant difference in pRR50 on comparing all three phases (p = 0.036). However, there was no significant variation in other parameters such as very low frequency (VLF, ms^2^), VLF (%), LF (ms^2^), LF (%), HF (ms^2^), HF (%), LF/HF, SDNN (ms), RMSSD (ms), Poincare plot standard deviation perpendicular to the line of identity (ms), Poincare plot standard deviation along the line of identity (ms), systolic blood pressure (mmHg), and diabolic blood pressure (mmHg) during, before, and after exposure to mobile phone calls. There was no significant difference in the value of all parameters between males and females (p < 0.05).

Conclusions

Mobile phone calls may influence HRV and autonomic balance. This change may be affected by the electromagnetic field and by speaking as well.

## Introduction

Nowadays, mobile phones are an important part of life used for calling, online studying, shopping, watching movies and videos, making videos and reels, paying bills, etc. The coronavirus disease 2019 (COVID-19) pandemic posed a global health crisis that affected more than 200 countries. During the outbreak of COVID-19, almost all countries implemented lockdowns and shut down public gatherings, which stopped in-person interaction with family and society. People were forced to use technologies such as mobile phones to reduce their distance as well as their anxiety. There is no doubt that mobile phones provide an easy way of communicating with colleagues, friends, and relatives. However, as we know, science is a double-sided sword, so every technology that provides such benefits comes with a set of negative impacts. Mobile phones emit electromagnetic waves in the range of 850-1,900 MHz [1]. These rays are absorbed by the body and affect the health of people in various ways [2,3]. The electrical activity of the heart and brain is also affected by electromagnetic field [4]. Signals produced by the operation of mobile phones likely interfere with implanted pacemakers. Exposure to electromagnetic field [5] has been shown to increase resting blood pressure.

Many authors have suggested that electromagnetic waves, which are emitted by mobile phones, affect autonomic functions, but the results are ambiguous [6]. The impact of long-term exposure to mobile phones on the autonomic nervous system has been reported in previous studies [7]. Very few studies have evaluated the effect of acute exposure to electromagnetic field [5] due to mobile phones on autonomic functions. Therefore, in this study, we aimed to determine the acute effect of cell phone calls on heart rate variability (HRV) and blood pressure. It was hypothesized from previous studies that parasympathetic activity increases after short-term exposure to mobile phones. The authors reported that due to the effect of the electromagnetic field, the observed changes in HRV were noted. but there may be some effect of speaking on these observed effects [8]. The time domain parameters in his study did not change significantly in all three periods. Three different methods for the analysis of HRV have been employed in most studies, namely, time domain, frequency domain, and non-linear methods [9].

## Materials and methods

Study settings

This analytical study was conducted among 30 subjects aged between 18 and 30 years after obtaining written consent from all participants. The study was approved by the Ethics Committee of Dr. Ram Manohar Lohia Institute of Medical Sciences (approval number: RC-139/20/RMLIMS/2020 dated 10.09.2020).

Study participants

Both male and female healthy subjects who were using mobile phones for more than five years with minimum daily usage of 30 minutes were included in the study. A brief history was obtained and a physical examination was performed to exclude any diseases. The subjects who had a history of any chronic disease, smoking, alcoholism, any medication affecting the autonomic nervous system, and obesity were excluded from the study. The subjects were asked to not consume caffeinated products and perform a physical activity of vigorous type a day before data collection. Data collection was done between 3.00 and 4.00 pm to avoid diurnal variation.

Data collection

HRV was recorded using a three-channel Physiograph (AD Instruments South Asia (India) Pvt. Ltd., New Delhi, India) with software LabChart PROV8.18 with HRV Module version 2.0.3 for 10 minutes. Blood pressure was measured from the left arm using Omron 7130L CP fully automatic digital blood pressure apparatus.

Patients were asked to lie comfortably in a quiet room with standard environmental conditions for 30 minutes and the following procedure was followed: Period I: Baseline HRV and blood pressure were recorded in the supine position for 15 minutes without a mobile phone (baseline recording). Period II: Participants were asked to hold their mobile phone (1,800/1,900/850/900 MHz GSM network with a Sar value of 0.290 W/kg) near their right ear for 15 minutes in silent mode while the investigator was calling it with another mobile. Blood pressure and HRV were recorded again during the call. Period III: Blood pressure and HRV were again recorded for 15 minutes after disconnecting the call and removing the mobile phone.

Statistical analysis

Data were expressed as mean ± SD. Comparison of parameters in all three phases was done by Student’s t-test and paired t-test using SPSS version 21 for windows (IBM Corp., Armonk, NY, USA). A p-value less than 0.05 was considered significant.

## Results

**Figure 1:**
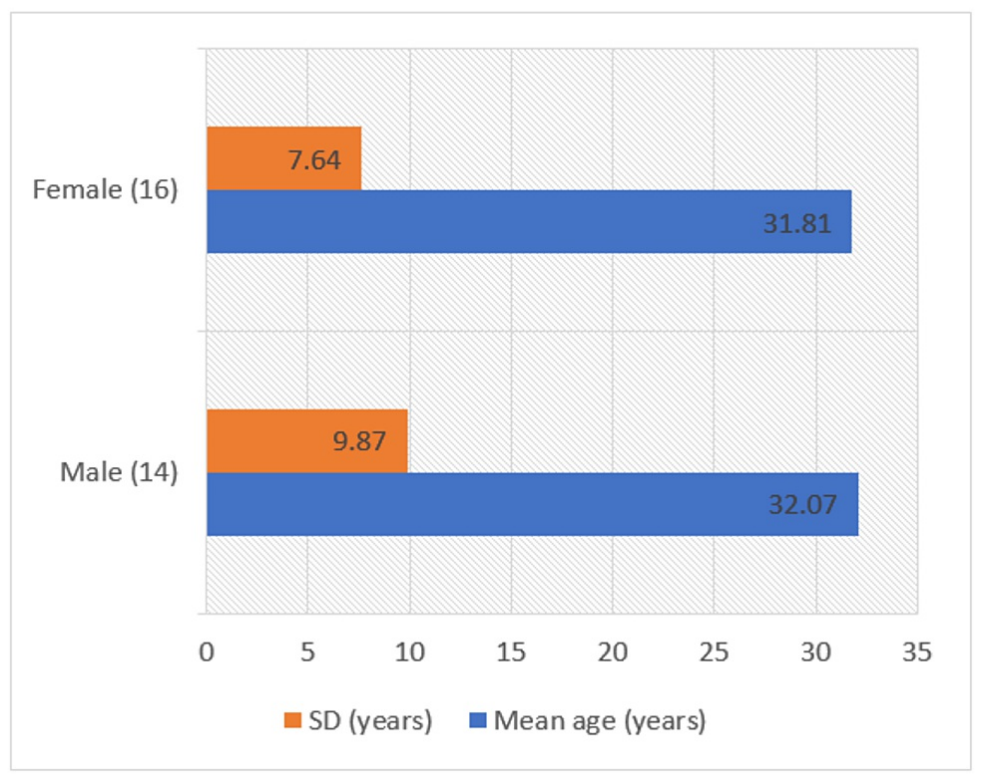
Gender and age distribution of participants.

A total of 30 subjects (14 males and 16 females) participated in the study. The mean age of participants was 31.93 ± 8.59 years (32.07 ± 9.87 years for males, and 31.81 ± 7.64 years for females). There was no statistically significant difference in the mean age of male and female subjects (p = 0.468) (Figure 1).

Table 1 shows the baseline parameters of HRV, heart rate, and blood pressure. Both time and frequency domain parameters were recorded. Time domain parameters were recorded in terms of the standard deviation of normal to normal interval (SDNN), root mean square of successive differences between normal heartbeats (RMSSD), R-R intervals greater than 50 ms (pRR50), and mean heart rate (MHR), and frequency domain parameters were recorded as total power, low-frequency power (LF), high-frequency power (HF), and the ratio of low-frequency to high-frequency power (LF/HF). There was no significant difference in the value of all parameters between males and females (p < 0.05).

**Table 1:** Baseline heart rate variability parameters and blood pressure. DBP = diastolic blood pressure; SDNN = standard deviations of normal to normal interval; RMSSD = root mean square of successive differences between normal heartbeats; pRR50 = R-R intervals greater than 50 ms, MHR = mean heart rate; TP = total power; LF (%) = low-frequency power percentage; HF (%) = high-frequency power percentage; LF/HF = ratio of low-frequency to high-frequency power; SD1 = Poincare plot standard deviation perpendicular to the line of identity; SD2 = Poincare plot standard deviation along the line of identity

Parameters	N = 30 (mean ±SD)	Males (n = 14) (mean ± SD)	Females (n = 16) (mean ± SD)	P-value
SDNN (ms)	43.29 ± 50.37	54.71 ± 71.82	33.48 ± 14.54	0.257
RMSSD (ms)	33.15 ± 53.25	45.89 ± 76.11	22.00 ± 13.34	0.226
pRR50	6.41 ± 12.27	6.41 ± 10.77	6.42 ± 13.80	0.997
TP (ms^2^)	1,516.95 ± 1,598.56	1,886.20 ± 2,041.84	1,193.85 ± 1,043.37	0.243
LF (%)	31.72 ± 16.20	37.22 ± 18.30	26.92 ± 12.81	0.082
HF (%)	23.41 ± 14.01	20.16 ± 9.18	26.26 ± 16.97	0.241
LF/HF ratio	1.91 ± 1.42	2.16 ± 1.53	1.70 ± 1.34	0.386
SD1	23.25 ± 37.70	32.03 ± 53.97	15.57 ± 9.45	0.239
SD2	54.67 ± 60.61	66.42 ± 86.56	44.39 ± 19.11	0.329
DBP (mmHg)	70.2 ± 7.56	72.79 ± 9.01	67.38 ± 6.44	0.066
SBP (mmHg)	99.93 ± 12.69	104.57 ± 10.62	95.88 ± 13.26	0.133
Mean heart rate (BPM)	88.85 ± 13.82	84.21 ± 10.72	92.91 ± 14.06	

As shown in Table 2, there was a significant difference in pRR50 when compared to all three phases (p = 0.036). However, there was no significant variation in other parameters such as very low frequency (VLF, ms^2^), VLF (%), LF (ms^2^), LF (%), HF (ms^2^), HF (%), LF/HF, SDNN (ms), RMSSD (ms), Poincare plot standard deviation perpendicular to the line of identity (SD1, ms), Poincare plot standard deviation along the line of identity (SD2, ms), systolic blood pressure (mmHg), and diabolic blood pressure (mmHg) during, before, and after exposure mobile phone calls.

**Table 2:** Changes in the parameters before, during, and after exposure to mobile phone calls. DBP = diastolic blood pressure; SDNN = standard deviations of normal to normal interval; RMSSD = root mean square of successive differences between normal heartbeats; pRR50 = R-R intervals greater than 50 ms; MHR = mean heart rate; TP = total power; LF (%) = low-frequency power percentage; HF (%) = high-frequency power percentage; LF/HF = ratio of low-frequency to high-frequency power; SD1 = Poincare plot standard deviation perpendicular to the line of identity; SD2 = Poincare plot standard deviation along the line of identity

Parameters	Before exposure to mobile phone calls (baseline) (mean ± SD)	During exposure to mobile phone calls (mean ± SD)	After exposure to mobile phone calls (mean ± SD)	P-value
DBP (mmHg)	69.90 ± 8.09	102.40 ± 12.52	98.27 ± 98.27	0.394
SBP (mmHg)	99.33 ± 13.38	72.33 ± 9.93	69.40 ± 8.47	0.440
SDNN (ms)	43.39 ± 50.38	45.96 ± 50.88	47.76 ± 59.22	0.951
RMSSD	33.15 ± 53.25	35.00 ± 57.95	34.97 ± 60.55	0.990
pRR50	6.42 ± 12.27	7.76 ± 14.78	7.76 ± 14.76	0.036^*^
MHR	88.85 ± 13.82	89.11 ± 2.48	87.53 ± 2.43	0.893
TP	1,516.95 ± 1,598.56	5,413.10 ± 2,0753.25	7,171.50 ± 31,538.51	0.592
LF (%)	31.72 ± 16.20	26.21 ± 13.00	32.63 ± 12.55	0.165
HF (%)	23.41 ± 14.01	22.52 ± 17.70	24.45 ± 16.78	0.857
LF/HF ratio	1.91 ± 1.42	2.09 ± 2.09	2.02 ± 2.02	0.900
SD1 (ms)	23.25 ± 37.70	24.76 ± 40.99	24.88 ± 42.84	0.985
SD2 (ms)	54.67 ± 60.61	59.24 ± 60.03	61.97 ± 72.56	0.907

## Discussion

According to the literature, the part of the nervous system that is responsible for homeostasis is the autonomic nervous system. The electromagnetic waves which are generated at the time of mobile phone use may influence the body’s homeostasis along with the autonomic nervous system, including HRV and blood pressure. HRV is the simplest, most sensitive, specific, reproducible, indirect measure of autonomic activity. Studies have examined the long-term effect of mobile phone usage on the autonomic nervous system. However, there is a lack of literature on the acute effects of the use of mobile phones, particularly on HRV. Hence, this study aimed to address this gap and identify the acute impact of mobile phone radiation on HRV and blood pressure.

In most studies, three different methods have been used to analyze HRV, including time domain, frequency domain, and non-linear methods [10]. In this study, we also followed previous studies and included all three methods of HRV analysis.

The study showed that the MHR slightly increased during the exposure and reduced after the exposure; however, no significant difference was observed in the three different phases. The MHR was within the normal range (60-100 beats per minute) in all three phases [11]. Similarly, DBP and SBP did not change significantly during and after exposure to mobile phones (p < 0.05). These findings were supported by previous studies which observed no change in the heart rate on electrocardiogram and blood pressure in all three phases [5,12]. The association between the use of mobile phones and changes in blood pressure and heart rate [13] was also not confirmed in previous studies.

The analysis of time-domain parameters of HRV in periods I, II, and III showed that pRR50, an indicator of parasympathetic activity, increased significantly during exposure to a mobile phone call and remained elevated after the call (p > 0.05). This finding differs from a previous study that reported a significant increase in SDNN during mobile phone calls than periods I and III. Another study also showed an increase in SDNN during a call with a mobile phone.

Our study did not show a significant change in the frequency parameters of HRV. However, previous studies found an increase in VLF and HF while a decrease in LF and LF/HF, indicating an increase in parasympathetic activity.

The pNN50 is closely correlated with parasympathetic activity and is a more reliable index than other parameters of time-domain analysis in short-term measurements of HRV.

Wilén et al. found sympathetic dominance during the use of a mobile phone; however, they compared the HRV of subjects who experienced mobile phone-related symptoms with the control group having no symptoms in response to psycho-physiological tests [14]. They correlated it with emotional stress because psycho-physiological tests may influence HRV. In our study, only healthy subjects were included.

We also compared the non-linear parameters of HRV, which showed no significant difference during all three periods. Type of exposure to the electromagnetic field in context with intensity and duration of exposure and other factors. For these reasons, the results of our studies may differ from those reported by other studies.

Our study has a limitation in that we did not include a control group; however, we compared HRV during exposure to a mobile phone call, with the baseline HRV of subjects before exposure serving as a control considering that the study participants were healthy volunteers. Attention should be paid to the health risks of subjects who are exposed to electromagnetic fields occupationally and by mobile phone use for a long time.

## Conclusions

Mobile phone calls may influence HRV and autonomic balance. Although this change may be affected by the electromagnetic field, some influence of speaking is not avoidable. Furthermore, because of global relevance, it is important to conduct a longitudinal study to find the long-term effects of acute exposure to electromagnetic fields produced by mobile on the autonomic balance.
